# Harnessing Immersive Virtual Reality: A Comprehensive Scoping Review of its Applications in Assessing, Understanding, and Treating Eating Disorders

**DOI:** 10.1007/s11920-024-01523-2

**Published:** 2024-07-31

**Authors:** Anna Flavia Di Natale, Silvia Francesca Maria Pizzoli, Giulia Brizzi, Daniele Di Lernia, Fabio Frisone, Andrea Gaggioli, Elisa Rabarbari, Osmano Oasi, Claudia Repetto, Chiara Rossi, Elisa Scerrati, Daniela Villani, Giuseppe Riva

**Affiliations:** 1 Research Centre in Communication Psychology, Università Cattolica del Sacro Cuore, Largo Gemelli 1, Milan, 20123 Italy; 2 Department of Psychology, Università Cattolica del Sacro Cuore, Largo Gemelli 1, Milan, 20123 Italy; 3 Applied Technology for Neuro-Psychology Lab, IRCCS Istituto Auxologico Italiano, Via Magnasco, 2, Milan, 20149 Italy; 4 Humane Technology Lab, Università Cattolica del Sacro Cuore, Largo Gemelli 1, Milan, 20123 Italy

**Keywords:** Immersive virtual reality, Eating disorders, Assessment, Treatment, Body image, Virtual exposure therapy, Body swap, Cue exposure

## Abstract

**Background:**

Immersive Virtual Reality (IVR) has shown promise in the assessment, understanding, and treatment of eating disorders (EDs), providing a dynamic platform for clinical innovation. This scoping review aims to synthesize the recent advancements and applications of IVR in addressing these complex psychological disorders.

**Methods:**

This review followed the Preferred Reporting Items for Systematic Reviews and Meta-analysis Protocols, focusing on studies published in the past five years. It included peer-reviewed papers that used IVR for ED assessment, examination, or treatment. A comprehensive database search provided a selection of relevant articles, which were then methodically screened and analyzed.

**Results:**

Twenty studies met the inclusion criteria, with a primary focus on Anorexia Nervosa (AN), Bulimia Nervosa (BN), and Binge Eating Disorder (BED). The application of IVR was categorized into three areas: assessment, understanding, and treatment. IVR was found to be an effective tool in assessing body image distortions and emotional responses to food, providing insights that are less accessible through traditional methods. Furthermore, IVR offers innovative treatment approaches by facilitating exposure therapy, modifying body-related biases, and enabling emotional regulation through embodied experiences. The studies demonstrate IVR’s potential to improve body image accuracy, reduce food-related anxieties, and support behavioral changes in ED patients.

**Conclusion:**

IVR stands out as a transformative technology in the field of EDs, offering comprehensive benefits across diagnostic, therapeutic, and experiential domains. The IVR’s ability to simulate the brain’s predictive coding mechanisms provides a powerful avenue for delivering embodied, experiential interventions that can help recalibrate distorted body representations and dysfunctional affective predictive models implicated in EDs. Future research should continue to refine these applications, ensuring consistent methodologies and wider clinical trials to fully harness IVR’s potential in clinical settings.

**Supplementary Information:**

The online version contains supplementary material available at 10.1007/s11920-024-01523-2.

## Introduction

Eating disorders (EDs) are complex psychopathological conditions characterized by abnormal eating habits and a distorted body image (BI), significantly affecting individuals’ physical health and psychosocial well-being. EDs include a broad spectrum of conditions, of which Anorexia Nervosa (AN), Bulimia Nervosa (BN) and Binge Eating Disorder (BED) are the primary types. AN is classified into two primary subtypes: the restrictive subtype, where individuals achieve extreme body weight through dieting, fasting, or excessive exercise; and the purging subtype, characterized by an unhealthy low body weight with episodes of binge eating followed by compensatory behaviors such as vomiting or the use of laxatives to prevent weight gain. BN is distinguished by recurrent episodes of binge eating accompanied by compensatory behaviors to counteract the effects of overeating and weight gain. In contrast, BED involves repeated episodes of binge eating without subsequent compensatory actions [1].

In Western countries, a substantial proportion of young women and men, up to 17.9%, meet the diagnostic criteria for an ED in early adulthood [2], and the number of diagnoses is dramatically increasing after Covid-19 [3]. What underscores these conditions as a mental health emergency is the increasing prevalence combined with the limited effectiveness of available prevention, assessment, and treatment procedures. This is partially linked to multifaceted factors that contributes to EDs etiology and maintenance.

EDs manifest primarily across two dimensions: behavioral and experiential. The behavioral domain encompasses the actions undertaken by individuals to exert control over their bodies, including, food elimination, food restriction, and episodes of binge eating. These behaviors are the visible manifestations of the disorder, observable and quantifiable. However, the complexity of EDs also reaches the experiential domain, namely the internal, subjective experiences of individuals with EDs. Specifically, the experiential domain includes the emotional, perceptual, and cognitive aspects that shape individuals’ relationships with food and their bodies, which in turn influence overall daily functioning.

On one side, EDs are characterized by profound food-related dysfunctional emotional reactions [4]. The fear of gaining weight, the anxiety or disgust triggered by certain foods in certain situations, and the guilt following eating are emblematic of the disordered relationship with food that characterizes EDs. This complex emotional experience significantly impacts the individual’s ability to maintain a healthy eating behavior, often leading to avoidance, restriction, compensatory or uncontrolled behaviors that further exacerbate the disorder.

On the other side, a critical and pervasive issue is BI distortion [5, 6], where individuals inaccurately perceive their bodies as larger or more flawed than reality and deeply link their self-esteem and self-worth to distorted BI. This distortion is deeply linked to a significant difficulty in developing a stable sense of self, exacerbated by a lack of coherent, first-person experience of their body [7]. Individuals with EDs often struggle to feel a direct, personal connection to their bodily sensations and emotions. Instead, they adopt an external, object-centered perspective (self-objectified) influenced by societal ideals and past observations [8].

Intriguingly, two separate longitudinal studies conducted over four years, each involving upwards of 2000 participants [9, 10] revealed that self-objectification held a significantly higher predictive value for both the remission and emergence of EDs when compared to other widely acknowledged factors - body dissatisfaction, thin-ideal internalization, negative affectivity and lower self-esteem - that demonstrated significant less predictive power. This suggests a potentially essential role for self-objectification in the progression and mitigation of EDs, meriting further investigation within the field.

Recently, the Allocentric Lock Hypothesis (ALH) proposed that EDs may arise from difficulties in multisensory body integration [11]. This integration process involves combining internal bodily signals (e.g. hunger, proprioception) with external sensory information (e.g. vision, touch) and autobiographical memories (e.g., remembering past experiences related to body image or eating, such as recalling past comments about one’s appearance or feeling shame during social eating situations) to form a coherent representation of one’s physical self [12].

According to predictive coding theories, the brain continuously generates and updates an internal model of the body and surroundings, allowing it to predict and integrate diverse sensory inputs through embodied simulations [13]. The ALH proposes that in EDs, there is a disruption in these predictive mechanisms underlying multisensory integration [11]. As a result, individuals with EDs become locked into an observer-based (allocentric) embodied simulation of their body [14, 15]. Negative experiences like teasing, objectification through social media exposure, or distorted autobiographical memories originally shape the allocentric perspective. This outdated model remains rigid, failing to update accurately despite contradictory information from current sensory inputs about the individual’s actual bodily state. This persistent allocentric representation can cause ongoing BI disturbances, leading to persistent dissatisfaction and shame. These experiences trigger anxiety and drive maladaptive behaviors aimed at controlling perceived bodily flaws based on memory rather than true corporeal reality.

In this context, a key advantage of Immersive Virtual Reality (IVR) for EDs is that it can simulate virtual bodies and the worlds around them that dynamically adjust to the user’s actions, providing predicted multisensory feedback just as the brain generates and updates internal body models to anticipate sensory inputs [16]. This is particularly supported by advanced IVR technologies, such as head-mounted displays (HMDs), that enable real-time interaction with the virtual world through motion tracking, hand controllers, and sensory feedback devices.This alignment makes IVR an unprecedented “playground” to directly apply principles of predictive coding.

Through carefully constructed virtual environments, it is possible a controlled delivery of challenging multimodal experiences (e.g., exposure to high-calorie food) that induce modest prediction errors in a safe, immersive manner [17]. For example, IVR can recreate triggering situations, such as mealtimes, in personalized environments, placing patients in a range of contexts from kitchens to restaurants, whether alone or with others, and presenting various types of food [18]. These environments have the potential to elicit patients’ authentic emotional reactions that allow clinicians to understand deeper the patient’s emotional difficulties and to work with them on managing the emotional responses.

Moreover, IVR allows the embodiment in bodies different from the actual one (e.g., full body illusions) providing disconfirming multisensory feedback, that create opportunities to update the internal body models of the user iteratively [19]. For example, by embodying virtual avatars that closely mirrors or subtly adjusts their physical form, users can experience a different perspective on their body size, shape, or appearance [20]. This “re-training” of the predictive mechanisms can foster more accurate modeling of reality with reduced predictive biases that contribute to ED psychopathology [21].

Initial review works have focused on outlining and summarizing the application of IVR in EDs [18, 22, 23], and preliminary studies suggest its efficacy in addressing core EDs symptoms [16]. These data suggest that IVR could be a supportive tool for clinical practice, as it can offer an alternative or an addition to available assessment and intervention approaches.

### Rationale

This scoping review aims to explore the current use of IVR technology across various aspects of eating disorders (EDs). This work moves beyond previous theoretical works, that either provided a general description of the techniques [23], or focused on specific techniques [20], specific EDs [18] or specific outcomes [24], aiming at providing a more updated and comprehensive representation of the field [22].

Specifically, it offers a detailed analysis of novel IVR methods in EDs assessment, understanding and treatment, evaluating their effectiveness across various disorders. Furthermore, it not only evaluates the effectiveness of IVR in key aspects of EDs, including body image distortion and food-related anxiety, but also discusses its integration with established treatments.

## Methods

### Protocol

The protocol of this scoping review was crafted following the Preferred Reporting Items for Scoping Reviews (PRISMA-ScR). By adopting this methodology, we aimed to provide an extensive analysis of the developments in IVR for EDs over the past five years, focusing on the range still not covered by other theoretical works [18, 22, 23].

### Eligibility Criteria

Data extraction was performed on March 30th, 2024. Peer-reviewed journal papers were included if they were published from January 1st, 2019, up to the extraction date, written in English, involved human participants, and described a measure for ED treatment or assessment using IVR. Both experimental and quasi-experimental studies were selected, including randomized controlled trials and pre-post design studies. Papers were excluded if they did not fit into the conceptual framework of the study or if they did not present empirical data (e.g., background articles). Papers focusing on non-immersive virtual reality were also excluded to maintain the focus on immersive VR experiences specifically.

### Information Sources

To identify potentially relevant documents, the following bibliographic databases were searched: Scopus, Web of Science, PsychInfo, Pubmed. The search strategies were drafted by A.F.D.N. and further refined through team discussion. The final search results were exported directly into Rayyan, and duplicates were removed by A.F.D.N.

### Search

The following string was used for the search: (“virtual reality” OR vr) AND (“eating disorder*” OR bulimia OR “binge eating” OR anorexia) and adapted for each database. Notably, the term “immersive” was intentionally omitted in favor of the broader term “VR” to avoid prematurely excluding relevant studies. This approach acknowledges that some research may refer to VR in a way that implicitly includes IVR, despite not combining the term “immersive” and “virtual reality” one after the other. The aim was to encompass a wider range of studies, subsequently refining the selection to focus on immersive experiences as needed, based on the context provided within individual papers. The final string for each database can be found in “Supplementary Materials”.

### Selection of Sources of Evidence

We performed a two-step screening of all the references generated by the search strategy. In step 1, two authors (A.F.D.N. and S.F.M.P.) independently screened the titles and abstracts to exclude any irrelevant references that did not meet eligibility criteria. Conflicts were resolved through discussion. In step 2, two authors (A.F.D.N., S.F.M.P.) independently screened the full texts of the references selected in Step 1 against the eligibility criteria. To minimize bias during the selection process, the screening was blind, using the option “blind status on’’ in Rayyan. Once all authors have completed the selection process, the blinding status was turned off, and any disagreement was resolved through consensus.

### Data Charting

The team developed a data-charting form to decide on the variables for extraction. Two authors (A.F.D.N., S.F.M.P.) independently gathered the data, then discussed their findings with the rest of the team, refining the charting form through an iterative process to ensure comprehensive data collection and analysis.

## Synthesis of Results

**Fig. 1:**
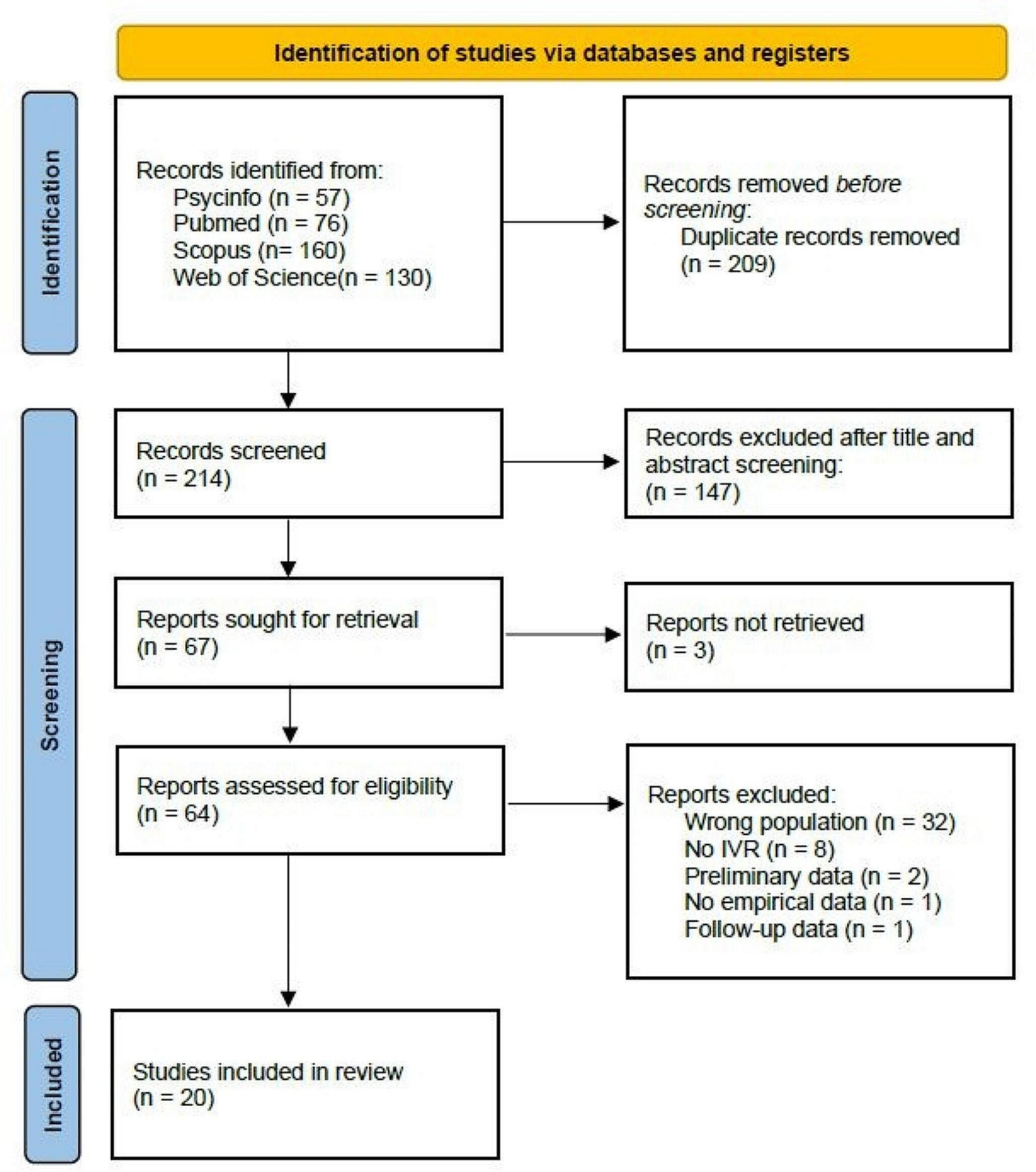
PRISMA 2020 flow diagram

A total of 423 papers were found. Two-hundred and nine were removed as duplicates and 147 were removed after screening Step 1 (titles and abstracts). A total of 67 were sought for retrieval and 3 were not found. As a result, a total of 64 full texts were screened for eligibility. After full text screening a total of 20 articles met the inclusion criteria and were included in our scoping review. The flowchart of paper selection is shown in Fig. 1.

Sixteen papers included participants with AN, 2 studies included patients affected by BED and only 1 study considered individuals with solely BN and 1 with BN and BED. Table 1 summarizes the sociodemographic information of the population of the included studies.

**Table 1:** Sociodemographic information

	First author	Year	Intervention group	Intervention sex	Intervention age	Control group	Control sex	Control age	n_tot
1	Ascione	2023	AN	23 F	15.30 ± 1.29	n.a.	n.a.	n.a.	23
2	Behrens (study 2)	2023	AN	20 F	30.1 ± 11.9	n.a.	n.a.	n.a.	20
3	Bektas	2023	AN	66 F, 4 M	25.7 ± 7	n.a.	n.a.	n.a.	70
4	Di Lernia	2023	AN	15 F	18.93 ± 3.97	HC	15 F	22 ± 0.59	30
5	Ferrer-Garcia	2021	AN	11 F, 2 M	n.s.	n.a.	n.a.	n.a.	13
6	Fisher	2020	AN	31	13.9 ± 4.25	n.a.	n.a.	n.a.	31
7	Malighetti	2020	AN	7 F	17 ± 1.87	n.a.	n.a.	n.a.	7
8	Malighetti	2021	BED	1 F	54	n.a.	n.a.	n.a.	1
9	Matamala-Gomez	2021	AN	5 F	17 ± 1.8	n.a.	n.a.	n.a.	5
10	Max	2023	BED	31 F, 7 M	36.26 ± 13.37	n.a.	n.a.	n.a.	31
11	Nameth	2021	BED + BN	11 F, 1 M	40.90 ± 15.7	n.a.	n.a.	n.a.	11
12	Natali	2024	AN	140 F, 2 M, 3 other	21.75 ± 6.68	n.a.	n.a.	n.a.	145
13	Porras-Garcia	2020 (37)	AN	1 F	14	n.a.	n.a.	n.a.	1
14	Porras-Garcia	2020 (36)	AN	30 F	17.73	HC	43 F	21.12	73
15	Porras-Garcia	2020 (35)	AN	1 F	15	n.a.	n.a.	n.a.	1
16	Porras-Garcia	2021	AN	14 F, 2 M	18.25 ± 1.30	AN	19 F, 2 M	19.21 ± 1.78	35
17	Provenzano	2020	AN	20 F	23.30	HC	20 F	23.85	40
18	Sansoni	2024	BN	12 F	29.25 ± 7.75	BN	12 F	28.83 ± 6.48	24
19	Serino	2019	AN	1 F	30	n.a.		n.a.	1
20	Schroeder	2024	AN	60 F	19.1 ± 4.88	HC	29 F	20.0 ± 3.87	89

Overall, the included articles comprise a total sample of 661 participants, (479 with AN, 75 with BN, or BED, and 107 healthy controls), aged 14 to 65. The female gender was the most represented (638 females, 10 males, 3 non binary or others). Most of the studies were conducted in Europe (Spain, Italy, Germany, and French), two were carried out in the United States, and one in the United Kingdom.

To systematize results and better understand the impact of new IVR approaches in EDs treatment, we pooled the results according to three mutually exclusive categories: (1) studies where IVR is used as a diagnostic or assessment tool to identify patients and/or assess their symptoms (*assessment*), (2) Studies where IVR is used to better analyze certain aspects or processes which characterize people with EDS that might shape IVR treatment efficacy for EDs (*understanding*), and (3) studies aimed at testing the efficacy of IVR treatment on ED symptoms (*treatment*). Although some papers could fall into multiple categories, such as understanding and treatment [25] or assessment and treatment [26], we have placed each of them in the most representative category, based on the main aim of the study. An overview of the included studies is available in Table 2 and an overview of the IVR technology and procedure used is detailed in Table 3.

**Table 2:** Overall information of the included studies

First author	Year	Aims and scopes	Study design	Main findings
**Assessment**				
Bektas	2023	To explore the correlations between food-specific disgust, eating disorder psychopathology, and food interaction in anorexia nervosa patients through different virtual kitchen scenarios using VR.	cross-sectional	Significant positive correlations were found between food-specific disgust and eating disorder symptom severity. The study highlights the potential clinical utility of addressing food-specific disgust in anorexia nervosa treatment.
Fisher	2020	To assess body image disorder (BID) symptoms in anorexia nervosa (AN) using virtual reality (VR) with standardized 3D avatars compared to traditional paper-based figure rating scales (FRS). The study hypothesized that VR would improve body image perception and evaluation among adolescents with AN due to the enhanced visual perception and immersive conditions provided by VR technology.	retrospective, cross-sectional	Adolescents with AN overestimated their own body size regardless of the assessment tool used (VR or paper-based FRS). BPI and body dissatisfaction did not significantly differ between the two methods. VR-based evaluation was correlated with psychometric parameters related to BID and body dissatisfaction. Head-tracking data revealed a longer engagement with avatars representing malnutrition and underweight states, indicating a preference for thinner body images among participants.
Matamala-Gomez	2021	To test the usability and UX of the VR-based protocol for patients with AN, specifically how a virtual body-swapping illusion can influence their perception of their own body.	pre-test and post-test design without a control group	The study found high levels of embodiment (ownership, agency, and self-location) when patients were embodied in a virtual body that resembled their real perceived body size. User experience, measured through embodiment scores, think-aloud technique, and patient interviews, indicated that patients with AN could benefit from using VR as a technological solution for assessing and modulating body image distortions
Provenzano	2020	To characterize and mitigate body image distortion in AN by employing virtual reality to assess body overestimation and dissatisfaction, examining the effectiveness of embodied virtual avatars in altering these perceptions.	pre-test and post-test design	Patients with AN exhibited higher body dissatisfaction without overestimating their body size. The study underscored the cognitive-emotional component of body image distortion as more severely altered in AN, noting that embodiment through virtual reality did not significantly ameliorate these distortions.
**Understanding**				
Di Lernia	2023	To objective of the study was to the influence of interoceptive metacognition and predictive Bayesian models on embodiment and body perception distortions in patients with AN compared to healthy controls.	cross-sectional	Patients with AN demonstrated a disconnect between interoceptive perception and metacognitive evaluation of these perceptions, showing a pattern where higher levels of interoceptive metacognitive beliefs were associated with greater embodiment and body perception distortions. This pattern, however, was partially recovered following the clinical rehabilitation program.
Ferrer-Garcia	2021	To assess the effectiveness of VR-BE incorporating body related attentional bias, in addition to TAU, for reducing FGW, BIDs, and other ED symptoms in patients with AN.	RCT	Post-intervention, the experimental group showed significantly reduced FGW and BIDs compared to the control group, suggesting VR-BE’s efficacy in addressing core AN fears and disturbances. Body-related Attentional Bias before therapy predicted less favourable clinical outcomes, such as a smaller reduction in FGW, BID, and a smaller increase in body appreciation after treatment.
Max	2023	To investigate cognitive processes underlying BED through embodied interaction with food in VR, examining the relationship between behavioral biases in manual interaction with food and BED psychopathology, eating behavior, impulsivity, and food craving.	RCT	The study identified a faster recognition and approach behavior towards food, followed by a slower collection of food compared to office tools. This pattern suggests an initial impulsive reaction to food stimuli, followed by a more cautious or aversive motivational process in handling food. No significant changes in behavioral patterns were observed after a clinical intervention aimed at reducing BED symptoms, and no direct relationships between behavioral biases and sample characteristics were detected.
Schroeder	2024	To explore the behavioral patterns associated with food avoidance in individuals diagnosed with AN-restrictive type. The goal was to employ a novel VR stopping task to investigate the trajectories of food avoidance behavior and to understand if food avoidance stem from either automatic, habitual responses or heightened inhibitory control abilities.	pre-test/post-test with a control group	The paper explores food avoidance in patients with AN-restrictive type using a VR stopping task, finding evidence of persistent food avoidance and heightened inhibitory control in these patients.
**Treatment**				
Ascione	2023	The study aimed to assess the efficacy of a single session of a new body-related ABMT that combines VR with eye tracking in patients with AN. The goals were to reduce body-related AB by balancing attention between weight and non-weight-related body areas and to reduce BD levels.	pre-test/post-test without a control group	A significant reduction in both the total fixation time on weight-related body parts and BD levels post-intervention, suggesting the potential clinical utility of ABMT in treating AN.
Behrens (study 2)	2023	To evaluate the potential of virtual reality (VR) exposure to healthy weight bodies as an adjunct treatment for anorexia nervosa (AN), focusing on its effects on fear of weight gain among patients with AN.	pre-test and post-test without a control group	VR exposure to healthy weight bodies was found to evoke high arousal in patients with AN and demonstrated a trend toward improving fear of weight gain after four sessions of exposure.
Malighetti	2020	To explore the effectiveness of VR in unlocking the allocentric memory of the body in patients with AN by combining autobiographical recall and body-swapping techniques to affect body size distortion and related negative memories.	pre-test and post-test design without a control group	Participants showed reduced negative emotions and body shame, increased body satisfaction, and more accurate body perception post-intervention, suggesting the potential of VR as a tool for assessment and treatment of body perception in AN.
Malighetti	2021	To evaluate the effectiveness of a novel ER-focused VR immersive intervention in reducing EE and improving emotion regulation among individuals with a history of binge eating disorder.	case study	Post-treatment assessments indicated a decreasing trend in emotional eating, emotion dysregulation, binge episodes, depression, and anxiety, alongside an increasing trend in self-esteem and confidence in eating and performing eating-related activities.
Nameth	2021	To assess the translation of VR-CET from controlled research settings to a real-world clinical setting, focusing on its feasibility, acceptability, and preliminary signals of effectiveness for patients with BED or BN who have experienced at least one binge episode per week.	pre-test and post-test design without a control group	The study found significant reductions in OBEs and high acceptability among both patients and therapists. The adoption of VR-CET in clinical settings appears feasible and acceptable, with preliminary evidence suggesting its effectiveness in reducing binge eating symptoms.
Natali	2024	To investigates the feasibility of utilizing positive mood induction or social support to augment the impact of virtual food exposure on food-related anxiety in patients with AN.	RCT	Findings reveal decreased anxiety levels in the positive mood condition compared to the baseline condition, shedding light on potential strategies to mitigate food-related distress in this population
Porras-Garcia	2020a	To assess the efficacy of VR Body Exposure Therapy in reducing FGW, BID, body-related anxiety, and improving BMI in a patient with AN.	case study	The intervention led to significant improvements in FGW, body-related anxiety, drive for thinness, and BID immediately after treatment, with a slight increase in BMI. Some improvements decreased at the 3-month follow-up.
Porras-Garcia	2020b	To assess the VR-based body exposure’s utility in eliciting FGW, body anxiety, and body-related attentional bias among patients with AN.	cross-sectional	Patients with AN experienced higher FGW, body anxiety, and attentional bias towards weight-related body areas than healthy controls. Interestingly, patients with AN showed lower level of FBI compared to healthy participants, suggesting a potential disconnect or discomfort with embodying a virtual body closely resembling their actual physique.
Porras-Garcia	2020c	To provide preliminary evidence of VR-BET’s usefulness in treating AN by reducing FGW, body-related anxiety, and dissatisfaction, and by achieving a healthy BMI.	case study	Significant reductions in FGW, drive for thinness, body-related anxiety, and dissatisfaction post-intervention, with BMI increasing to healthy levels. These improvements were largely maintained at a 5-month follow-up, except for FGW.
Porras-Garcia	2021	To evaluate the efficacy of virtual reality-based body exposure therapy (AN-VR-BE) combined with treatment as usual (TAU) for reducing fear of gaining weight (FGW), body image disturbances (BIDs), and other ED symptoms in patients with AN.	RCT	The study found that AN-VR-BE combined with TAU was more effective than TAU alone in reducing FGW, body distortion, and body dissatisfaction among patients with AN. Improvements were significant for body distortion and body dissatisfaction, with trends toward improvement in FGW and other ED measures. These effects were maintained or became more pronounced at the three-month follow-up.
Sansoni	2024	To compare the effectiveness of a VR-CBT intervention to an Inpatient Program (IP) for female patients diagnosed with BN	RCT	The study found that the VR-CBT intervention resulted in a consistently stable BMI trajectory over time, contrasting with the control group’s BMI fluctuations. Additionally, VR-CBT demonstrated effectiveness in alleviating preoccupation with weight, fear of weight gain, and occurrences of binge eating and purging behaviors. Conversely, the IP group experienced a notable BMI increase post-treatment, followed by a decline between 9 and 12 months after discharge.
Serino	2019	To assess the potential of VR-based body swapping illusion in treating body-size distortion in AN by altering the patient’s body representation through multisensory bodily integration.	case study	The VR full body illusion was successful in monitoring and partially modifying the patient’s distorted body representation. It highlighted the importance of multisensory integration in the treatment of AN, suggesting the VR-based approach could serve as both an assessment and therapeutic tool.

**Table 3:** Intervention details

	First author	Year	VR technique	Technology	Procedure of VR-based intervention	Control group
1	Ascione	2023	VR-ABMT	HTC Vive Pro Eye head-mounted display with built-in Tobii eye tracker; VR environment developed on Unity 3D software.	Participants, embodied in a virtual avatar matching their physical characteristics, completed the ABMT in a VR setting. This involved directing attention to geometric figures appearing on various body parts of the avatar to balance attention across weight and non-weight-related body areas.	n.a.
2	Behrens	2023	VR-BE	Valve Index headset and controllers, VIVE trackers, Lenovo Legion Y740 laptop	Participants underwent four 30-min VR exposure sessions to a body of higher weight, aiming to elicit and manage fear of weight gain. The VR environment featured a u-shaped changing cabin with two virtual mirrors showing the avatar from front, side, and first-person perspectives.	n.a.
3	Bektas	2023	VR-FE	Oculus Quest 2 headsets with Unity3D game engine and Oculus Integration SDK.	Participants were exposed to one of three VR kitchen scenarios (kitchen only, kitchen plus virtual pet, and kitchen plus avatar), where they interacted with virtual foods. This setup was used to measure food-specific trait and state disgust, eye gazes towards, and touching of virtual foods, before and after VR exposure.	n.a.
4	Di Lernia	2023	VR-BS	Oculus Rift DK2, connected to a portable computer for the virtual reality illusion. The illusion was facilitated by synchronous visuotactile stimulation.	Participants experienced a VR full-body illusion in both synchronous (experimental) and asynchronous (control) conditions to assess the effect on body perception. The procedure included body size estimation tasks and embodiment questionnaires, performed at two time points: upon hospital admission and after a 12-week rehabilitation program.	Healthy controls underwent the same VR intervention in a single experimental session, allowing for comparative analysis with patients with AN.
5	Ferrer-Garcia	2021a	VR-BE	The study utilized HTC-VIVE head-mounted displays, VR controllers, and body trackers for full-body tracking, alongside a FOVE VR headset with eye-tracking for certain assessments.	The experimental group underwent AN-VR-BE, involving immersion in a VR environment where patients were exposed to a virtual body matching their real body size and then gradually introduced to a healthy BMI increase across sessions.	The control group received the standard treatment without the VR-based body exposure sessions.
6	Ferrer-Garcia	2021b	VR-BE	VR system including HTC-VIVE head-mounted display (HMD), VR controllers, body trackers, and a FOVE VR-HMD with eye-tracking technology. Powerful VR-ready computers with high-end CPU processors and graphic cards were used to run 3D immersive environments.	Participants in the experimental group received five sessions of AN-VR-BE, involving exposure to a virtual body matching their real-size silhouette and BMI. Small BMI increases were applied over successive sessions until reaching a healthy BMI target. The intervention aimed to reduce FGW by allowing patients to confront their body image fears in a controlled, virtual environment. Procedures included visuomotor and visuo-tactile stimulation to elicit the Full Body Illusion (FBI) of owning the virtual body.	Control group received treatment as usual (TAU) only.
7	Fisher	2020		Oculus Rift headset for VR exploration; VR software developed by C2CARE under the UNITY 3D framework; head-tracking technology for implicit measurement of explicit choices.	Participants evaluated their perceived and desired body figures using both paper-based FRS and VR-based avatar rating scale. In VR, participants engaged with ten 3D standardized female avatars arranged in a circle for a 15-minute session, during which they were asked to choose avatars that best represented their current and desired body forms. The head-tracking software calculated the time spent looking at each avatar (Fixation Time Percentage - FTP).	Comparison made with paper-based FRS.
8	Malighetti	2020	VR-BS	VR intervention included a head-mounted display connected to a laptop and a motion tracker (Microsoft Kinect sensor), immersing participants in a virtual environment and embodying them in a virtual avatar.	The intervention consisted of four sessions with VR embodiment from both egocentric and allocentric perspectives. The BMI of the avatar was progressively increased to normal weight across sessions. Participants recalled negative and positive life events to associate emotions with body sizes and estimated their real and ideal BMI using VR body size estimation tasks.	n.a.
9	Malighetti	2021	VR-CBT	The intervention was delivered using a stand-alone head-mounted display (HMD), specifically the Oculus Go.	The treatment comprised an initial assessment session, followed by two sessions focusing on emotion regulation and four sessions on emotional rescripting. Techniques included mindfulness-based strategies for exploring landscapes in VR to identify emotional states, immersive metaphorical journeys, and anchoring positive emotions to real-life experiences.	n.a.
10	Matamala-Gomez	2021	VR-BS	Virtual Reality (VR) with a head-mounted display (Oculus Rift), connected to a laptop and a Kinect Sensor for motion tracking.	The VR-based intervention involved embodying participants in a virtual avatar from both first-person and third-person perspectives, across four sessions. The technique aimed to create a body swap illusion, allowing participants to experience ownership, agency, and self-location in a virtual body that mirrors their perceived real body size. This intervention focused on modifying participants’ long-term memory of their body through visuomotor synchronization and autobiographical recall within the VR environment.	n.a.
11	Max	2023	VR-FE	Oculus Rift CV1 head-mounted display with Leap Motion sensor for real-time hand tracking, allowing participants to interact with virtual stimuli through actual hand movements.	Participants completed practice trials followed by the main task, where they had to recognize and collect one of two simultaneously presented objects (food vs. office tools), with the task designed to measure both the speed of recognition/approach and the collection of food objects compared to office tools.	n.a.
12	Nameth	2021	VR-CET	Oculus Rift head-mounted displays (HMD), Oculus sensors, and Oculus controllers were used to provide immersive VR experiences simulating real-life triggering eating-related situations.	The intervention involved up to eight one-hour sessions, starting with an assessment phase followed by an intervention phase where patients were exposed to virtual environments and food cues designed to provoke cravings and urges to eat without allowing actual binge eating.	n.a.
13	Natali	2024	VR-FE	Oculus Integration SDK for Unity was installed on Oculus Quest 2 headsets to visualize the VR kitchen environment. Participants experienced full immersion in a room-scale environment, facilitated by two Oculus Touch controllers for interaction with user interface menus and scene objects.	The study examines the potential of two interventions—positive mood induction and social support—to enhance the effectiveness of virtual food exposure in alleviating food-related anxiety. Participants were assigned to one of three conditions: virtual food exposure alone (baseline condition), virtual food exposure combined with positive mood induction (positive mood condition), or virtual food exposure combined with social support (social support condition). In the social support condition, an avatar provided participants with supportive and motivational dialogue aimed at empowering them to confront the voice of their eating disorder.	n.a.
14	Porras-Garcia	2020a	VR-BE	VR head-mounted display (HTC-VIVE) with full body motion tracking using additional body trackers.	The patient underwent five VR exposure sessions, starting with an avatar matching the patient’s current BMI. The BMI of the avatar was gradually increased in each session to simulate weight gain towards a healthy BMI. The intervention aimed to reduce FGW, body-related anxiety, and BID by allowing the patient to experience a full-body illusion (FBI) of a healthier body.	n.a.
15	Porras-Garcia	2020b	VR-BE	HTC-VIVE head-mounted display with full-body motion tracking and FOVE-Eye Tracking for eye movement detection	Participants owned a virtual body resembling their silhouette and BMI. The FBI over the virtual body was induced through visuo-motor and visuo-tactile stimulation. Subsequently, FBI, FGW, body anxiety, and body-related attentional bias towards weight-related and non-weight-related body areas were assessed.	Healthy college women divided into those with low and high body dissatisfaction.
16	Porras-Garcia	2020c	VR-BE	HTC-VIVE HMD with body trackers and FOVE Eye Tracking for gaze detection and registration. Virtual avatars created with Unity 3D and Blender 2.78.	Five sessions of VR body exposure therapy were conducted, starting with the patient’s current BMI and progressively increasing the BMI of the avatar across sessions. The therapy aimed to systematically expose the patient to her virtual silhouette in a mirror within the VR environment, with body parts that produced anxiety being illuminated and focused upon	n.a.
17	Provenzano	2020	VR-BE	Virtual reality environments created with 3D modeling software, presenting participants with personalized avatars to embody their perceived body size, and avatars representing a verisimilar loss and gain of their original weight.	Participants were exposed to personalized avatars through visuo-tactile stimulation, aiming to induce embodiment and assess changes in body image distortion. The study evaluated embodiment, body dissatisfaction, and emotional response to different-sized avatars.	The healthy control group was subjected to the same VR intervention procedure to compare body image distortion, embodiment, and emotional responses between patients with AN and individuals without eating disorders. This comparison aimed to elucidate the specific effects of VR body exposure therapy on the cognitive-emotional components of body image distortion in AN.
18	Sansoni	2024	VR-CBT	During VR-CBT sessions, participants wore a head-mounted display through which scenarios were presented. Utilizing the NeuroVR software, participants engaged with fourteen virtual critical environments.	Participants attended five weekly CBT-group sessions and underwent ten biweekly one-hour VR sessions utilizing NeuroVR software, featuring diverse virtual environments.	The group received five-week treatment periods, during which two licensed psychotherapists conducted CBT sessions.
19	Serino	2019	VR-BS	The protocol utilized a head-mounted display (HMD Oculus Rift DK2) connected to a portable computer, with a Razer Hydra motion-tracking device for inducing multisensory stimulation.	The patient underwent three sessions of the VR-based body swapping illusion, incorporating synchronous and asynchronous visuotactile stimulation to induce ownership over a virtual body. The intervention was designed to assess and potentially alter the patient’s body representation disturbances.	n.a.
20	Schroeder	2024		A mobile VR task was developed for the stand-alone Meta Quest 2 head-mounted display by the research team. The Meta Quest 2 controller was utilized within the virtual environment, depicted as a white right hand, with its position and movement continuously updated at the refresh rate.	The methodology involved investigating behavioral trajectories of food avoidance using a novel kinematic task in VR to record spatial displacement in stop-and go-trials to virtual food and control objects. Inhibitory control abilities were assessed through stopping performance, and habitual avoidance of virtual food was measured across both go-and stop-trials.	The healthy control group was subjected to the same VR intervention procedure

### Assessment

All studies included in this category explored how IVR can be employed to assess specific characteristics of AN. Four studies, among those identified, fell into this category. Specifically, three studies used body-swapping procedure and avatars to assess perceptual and affective BI disturbance [21, 26, 27], while one study used virtual food exposure (VR-FE) to evaluate emotional reactions to food related stimuli [28].

Fisher and colleagues [27] assessed BI (i.e., body distortions and body dissatisfaction) in AN patients with avatars or paper-based rating scales. The authors asked patients to select the Fig. (3D avatar vs. 2D image) that better fitted their real and ideal body shape, finding that the virtual avatar that patients selected corresponded closely to the silhouette they chose on a paper-based rating scale, thus yielding comparable self-rating results.

Provenzano and colleagues [26] used IVR to assess and measure the perceptual and cognitive-emotional components of BI disturbance in patients with AN. Through the creation of personalized avatars that vary in size, participants’ perceptions of their body size and shape were assessed. This allowed for a detailed assessment of how patients perceived their own body size (real body) versus how it actually appeared (ideal body), which is crucial for understanding the perceptual components of BI distortion in patients with AN. Although primarily designed as an assessment tool, the study also observed the influence of inducing a body ownership illusion on body dissatisfaction and perceptual distortions among patients with AN. Specifically, the authors observed that patients expressed more negative emotions after being embodied in the fattest avatar, especially in patients with more severe symptoms. This finding suggests that IVR can not only assess BI disturbances but also potentially influence them, indicating a dual role for IVR in both evaluating and addressing BI issues in therapeutic setting.

Similarly, Matamala-Gomez and colleagues [21] explored the use of IVR to modulate BI disturbances through a pilot study focusing on user experience. In their study, patients with AN were embodied in virtual avatars reflecting their perceived body size, and high levels of embodiment were reported. The results emphasized that immersive experiences with a high degree of body ownership can significantly affect how patients perceive their bodies, highlighting IVR’s potential to assess BI distortions in therapeutic settings.

As concerns VR-FE, Bektas and colleagues [28] used IVR to measure and assess food disgust in AN patients. The authors presented patients with a virtual kitchen, with or without other avatars. Results revealed a correlation between food disgust and patients’ symptoms severity, regardless of the presence of avatars in the virtual scenario, showing the clinical potential of IVR for in-depth exploration of food disgust in patients with AN.

### Understanding

Four studies focused on examining cognitive and emotional processes and variables relevant to the treatment of EDs in IVR. Two studies assessed cognitive processes related to BI in samples of individuals with AN [29, 30], while two explored the impact of interacting with foods during VR-FE in BED [31] or food avoidance in AN patients [32]. Ferrer-Garcia and colleagues [29] investigated body-selective visual attention bias and how it influenced clinical outcomes in patients with AN undergoing VR body exposure therapy (VR-BE). They observed that higher pre-intervention body-related attentional bias predicted less favorable clinical outcomes. Di Lernia and colleagues [30] used VR-induced body illusions to deepen the link between interoception and body distortions in patients with AN. Participants with AN exhibited greater interoceptive metacognition compared to healthy participants. The authors concluded that the presence of a strong interoceptive metacognition might impair the possibility of revising bodily distorted expectations with real-time multisensory bottom-up bodily information during IVR treatment. Max and colleagues [31] studied the presence of dysfunctional interaction patterns in the interaction with virtual foods in BED, revealing pathology-specific patterns in manual interaction with food (i.e., slower picking up of food items compared to non-food items), even though no significant link between interactions and behaviors or BED features emerged. Finally, [32] Schroeder and colleagues studied the phenomenon of food avoidance among individuals diagnosed with AN-restrictive type, compared with healthy controls, by employing a IVR stopping task. Results showed that AN patients had both persistent food avoidance tendencies and increased inhibitory control. Indeed, individuals with AN-R consistently displayed patterns of avoiding food, alongside an enhanced ability to exert inhibitory control over their eating behaviors compared to healthy participants.

### Treatment

Twelve of the included studies aimed to investigate the effectiveness of VR in treating EDs. They used VR-BE or body swapping (VR-BS) [25, 33–37], food exposure [38, 39], attentional bias training [40], or CBT combined with IVR [41] or psychological rescripting approaches combined with VR-BS to modify emotions and cognition [42].

Four studies utilized body exposure approaches with patients with AN. In a single case study on a patient with AN, Porras-Garcia and others [35, 37] observed that seeing one’s body from an external (observer) perspective diminished negative beliefs associated with the “self” and enhance body acceptance. They also noted that VR-BE therapy facilitated an increase in BMI reaching healthy levels and reduced fear of gaining weight (FGW); moreover decrease in body-related anxiety, drive for thinness, and BI disturbances were maintained after 5 months. A subsequent RCT with the same approach [43] revealed that patients with AN in the VR-BE group exhibited significant reductions in FGW and BI disturbances compared to the control. The exposure to healthy-weight virtual bodies was confirmed to elicit body concerns and treat and diminish FGW in the study by Behrens and colleagues [33], suggesting that VR-BE could be an effective technique in AN treatment. Three studies focused on VR-BS approach in AN. VR-BS sessions, integrated into a multidisciplinary AN treatment, influenced a patient’s body perception and multisensory body integration in Serino and colleagues [25], while the combination of autobiographical recall techniques and VR-BS was pilot-tested by Malighetti and colleagues [34], showing reductions in negative emotions and body shame, improvements in body satisfaction, and accuracy of body perception after VR sessions. Furthermore, the with a virtual avatar matching one’s BMI proved to be a useful intervention tool, as it resulted in a reduction of fear of weight gain, body anxiety, and attentional bias towards weight-related body parts in patients with AN compared to healthy controls, according to Porras-Garcia and colleagues [36].

Concerning VR-FE studies, a good feasibility and acceptability in treating BED with IVR cue exposure therapy in clinical settings emerged, with promising preliminary results in reducing binge episodes in the short-term and loss of benefits at follow-up [38]. While concerning AN patients, Natali and colleagues [39] (REF) investigated whether positive mood induction or social support can amplify the effect of VR-FE on food-related anxiety. Results showed that participants in the positive mood condition experienced lower levels of anxiety compared to baseline. Specifically, they reported reduced virtual food-related anxiety after exposure, with a medium effect size.

Concerning eye-movements and attentional aspects, two studies proved the effectiveness of IVR-based attention modification training with eye-tracking to reduce body-related attention bias and levels of body dissatisfaction in adolescents with AN [40].

Finally, the positive effect of combining traditional psychological treatments with IVR for BN and BED patients emerged in two papers. Specifically, CBT treatments and IVR for BN patients resulted in a more consistent BMI over time compared to the standard treatment, where BMI fluctuated significantly, and reduced preoccupation with weight, fear of weight gain, and instances of binge eating and purging [41], while combining VR-BS with the reshaping of emotional experience in immersive environments led to an improvement in emotion regulation and binge episodes [42]. These findings suggest that these interventions may enhance therapeutic outcomes and contribute to overall patient well-being.

## Discussion

The present scoping review aimed to investigate the use of IVR in addressing the complex nature of EDs. IVR tools resulted useful to target particularly the emotional, perceptual and cognitive aspects characterizing the experiential domain of EDs symptoms. Within the papers included in the review, three categories of IVR utilization were identified (*assessment*,* understanding*,* and treatment*).

Concerning IVR as an *assessment tool*, studies revealed that immersive experience can efficiently assess both perceptual and cognitive-emotional BI components [26–28, 44]. This aligns with previous research [30, 45], suggesting that IVR can provide ecological, reliable, and engaging assessment tools [46]. The latter aspect is critical in disorders such as AN, in which building therapeutic alliances and having genuine responses is often complicated [47]. Thus, technology offers advantages for both the clinician (i.e., accurate measurements less subject to bias) and the patient (i.e., active participation, engagement). Furthermore, following the trend of personalized medicine, IVR allows the development of tools tailored to patients’ needs, such as exposure to customized bodies [26] or specific foods [48].

As regards the *understanding* of the complex processes and features that characterize the psychopathology of EDs, the multisensory capabilities of IVR have emerged as a promising tool for enhancing our understanding of both inner and outer body perceptions [49]. In particular, the present scoping review confirmed the potential of eye-tracking integrated IVR tools in understanding attentional biases in EDs, which already emerged in a previous systematic review [20, 22].

The use of IVR as a *treatment* tool aligns with previous systematic reviews [22, 23, 50]. IVR has indeed demonstrated considerable promise in modifying the maladaptive behaviors characteristic of EDs. The studies included in this review consistently highlight IVR’s efficacy in creating realistic, controlled environments that facilitate BI retraining, exposure therapy, and cognitive restructuring within a clinical setting. IVR environments can indeed trigger genuine physiological and psychological responses like those experienced in real-world situations, providing a powerful platform confronting fears and practicing coping strategies [48]. Going a step further, we tried to understand why IVR might be so impactful by thinking about IVR features.

First, by experiencing VR-BS and VR-BE with avatars that represent either their actual or healthier BMI, patients are exposed to new perspectives on their BI. This exposure is crucial as it helps recalibrate the brain’s internal models of the body by providing real-time, accurate body-related sensory feedback, encouraging the brain to update these internal predictions, often reliant on distorted body internal representation [6]. In particular, IVR allows users to ‘change’ their bodies in unexpected ways, leading to discrepancies between expected and actual sensory feedback (prediction errors). These prediction errors can prompt significant updates in the brain’s internal body model^51^ , which may result in a more accurate body perception and healthier body interactions. This process can be further supported and enhanced by integrating autobiographical rescripting techniques, which help reshape personal experiences and improve emotion regulation in patients [34].

Second, the use of IVR in food exposure therapy has been particularly effective. By simulating environments that trigger disordered eating behaviors, such as settings that involve food interaction, IVR allows patients to navigate their anxieties in a safe and controlled manner. This method has been beneficial for patients with both AN and BED helping them to manage their fear of eating and reduce binge-eating episodes, respectively. Considering the efficacy of food exposure in IVR and the foundational role of prediction error in the treatment of EDs, as demonstrated in studies involving body illusions, future research might explore integrating food exposure with the deliberate creation of prediction errors. For example, combining food exposure with the predictive coding framework could involve altering expected sensory characteristics of foods within VR environments. This approach has the potential to induce unexpected discrepancies between anticipated and actual sensory feedback.

Considering both bodies focused approaches (VR-BS and VR-BE) and food exposure (VR-FE), IVR is distinguished as a deeply experiential and embodied technology. It enables direct modifications of perceptions, emotions, beliefs, and memories, bypassing the need for patients to consciously recall these elements or rely on metacognitive skills, which are often impaired in individuals with EDs.

In conclusion, this scoping review highlights the potential of IVR as a complementary tool in clinical practice in addressing the experiential domain that characterizes EDs. In this respect, it is crucial to emphasize that IVR should be viewed as an adjunctive resource rather than a standalone treatment. The primary benefit of IVR lies in its ability to offer a multisensory, embodied, and cognitive experience. This rich, immersive experience can significantly influence the brain’s predictive mechanisms related to body perception and emotional responses to key stimuli. However, it should be used judiciously and in conjunction with established therapeutic interventions to ensure a holistic and effective assessment and treatment approach for those suffering from EDs.

### Limitations

It is important to interpret the reported results considering some limitations. Firstly, there are few randomized controlled trials among the included studies. Additionally, many studies have small sample sizes, limiting statistical power and potentially introducing bias. Furthermore, the lack of standardized treatment protocols or consistent follow-up assessments across studies impacts the possibility to conclude on interventions efficacy. Moreover, male participants are underrepresented in these studies, which may influence the applicability of the findings to this population. Further research with larger and more diverse samples, as well as the implementation of standardized protocols, is needed to enhance the reliability and validity of IVR applications to EDs.

Furthermore, while the integration of IVR into clinical protocols for EDs holds great promise, it is necessary to consider potential side effects to ensure safe and effective implementation. Firstly cybersickness, a form of motion sickness originating from the sensory mismatch between visual input and physical movement, can be an issue for some patients, potentially exacerbating discomfort and hindering treatment progress. However, the current implementations of food exposure and body swap in IVR do not involve movement; patients remain stationary, which significantly limits the occurrence of cybersickness. Also, with the technical advances in IVR tools and the development of more sophisticated devices, this risk should be minimal, but is important for clinical centers to consider using devices that minimize this risk. Secondly, the realism of VR content, while creating authentic experiences, might induce stress or anxiety in patients, particularly during exposure to food-related stimuli. Tailoring treatments to individual patient needs or establishing a hierarchy of stimuli, as in the case of food exposure, can help mitigate these adverse effects. Implementing a hierarchy of stimuli and adjusting it based on the patient’s responses can help reduce anxiety and ensure a more comfortable and effective therapeutic experience. Lastly, the successful application of IVR in ED treatment necessitates specific training for healthcare professionals. Clinicians may lack the necessary expertise to effectively use this technology, highlighting the importance of comprehensive training programs. These programs should cover various aspects, including the technical operation of IVR equipment, understanding the therapeutic protocols, and being able to troubleshoot common issues. Additionally, training should emphasize strategies for managing possible side effects, such as cybersickness or anxiety induced by realistic VR content. By equipping clinicians with this practical knowledge, we can prevent misuse, optimize patient outcomes and make clinicians confident in using technological tools and managing possible side effects.

## Conclusion

In summary, the IVR’s ability to simulate embodied experiences that align with the brain’s predictive coding mechanisms provides a powerful avenue for delivering immersive, experiential interventions that can help recalibrate distorted body representations and affective predictive models implicated in eating disorders. This innovative approach holds significant potential for more effective and engaging treatments, particularly through their ability to immerse patients in experiences that directly address and integrate the experiential domain into comprehensive treatment plans. By offering innovative ways to confront and alter distorted BI and food-related negative reactions, IVR technology holds the potential to significantly impact the landscape of ED treatment, making it a valuable tool in the quest for more effective therapeutic interventions.

## Electronic Supplementary Material

Below is the link to the electronic supplementary material.

Supplementary Material 1

## Data Availability

No datasets were generated or analysed during the current study.
