# Correction: Harnessing Immersive Virtual Reality: A Comprehensive Scoping Review of its Applications in Assessing, Understanding, and Treating Eating Disorders

**DOI:** 10.1007/s11920-024-01563-8

**Published:** 2024-11-12

**Authors:** Anna Flavia Di Natale, Silvia Francesca Maria Pizzoli, Giulia Brizzi, Daniele Di Lernia, Fabio Frisone, Andrea Gaggioli, Elisa Rabarbari, Osmano Oasi, Claudia Repetto, Chiara Rossi, Elisa Scerrati, Daniela Villani, Giuseppe Riva

**Affiliations:** 1 Research Centre in Communication Psychology, Università Cattolica del Sacro Cuore, Largo Gemelli 1, Milan, 20123 Italy; 2 Department of Psychology, Università Cattolica del Sacro Cuore, Largo Gemelli 1, Milan, 20123 Italy; 3 Applied Technology for Neuro-Psychology Lab, IRCCS Istituto Auxologico Italiano, Via Magnasco, 2, Milan, 20149 Italy; 4 Humane Technology Lab, Università Cattolica del Sacro Cuore, Largo Gemelli 1, Milan, 20123 Italy

**Correction: Current Psychiatry Reports (2024) 26:470-486**

10.1007/s11920-024-01523-2

In the original version of this article, the authors Silvia F.M. Pizzoli and Giulia Brizzi should be affiliated to “Department of Psychology, Università Cattolica del Sacro Cuore, Largo Gemelli 1, Milan 20123, Italy” and “Humane Technology Lab, Università Cattolica del Sacro Cuore, Largo Gemelli 1, Milan 20123, Italy”.

The original article has been corrected.

