# Supplementary material for: Harnessing Immersive Virtual Reality: A Comprehensive Scoping Review of its Applications in Assessing, Understanding, and Treating Eating Disorders
Source: Curr Psychiatry Rep. 2024 Jul 31;26(9):470–86. doi: 10.1007/s11920-024-01523-2 (PMC11344702; doi:10.1007/s11920-024-01523-2)
Supplement: Supplementary file 1 — Supplementary Material 1 [file 11920_2024_1523_MOESM1_ESM.docx]

**Supplementary Materials. String used for search in each database**

| **Database** | **Search String** |
| --- | --- |
| Scopus | TITLE-ABS-KEY ( ( ( "virtual reality" OR vr ) AND ( "eating disorder*" OR bulimia OR "binge eating" OR anorexia ) ) ) |
| Web of Science | TS=(( "virtual reality" OR vr ) AND ( "eating disorder*" OR bulimia OR "binge eating" OR anorexia ) ) |
| PsychInfo | noft((("virtual reality" OR vr) AND ("eating disorder*" OR bulimia OR "binge eating" OR anorexia))) |
| Pubmed | (("virtual reality"[Title/Abstract]) OR (VR[Title/Abstract])) AND (("eating disorder*"[Title/Abstract]) OR (anorexia[Title/Abstract]) OR (bulimia[Title/Abstract]) OR(binge eating[Title/Abstract])) |
